# Integrating ACP into Nigeria’s culture and healthcare system for improved end-of-life care

**DOI:** 10.1017/S1478951525000197

**Published:** 2025-04-02

**Authors:** Rizky Andana Pohan, Dian Oktary, Riza Amalia, Rikas Saputra, Samudra Mutiara Hasanah, Adellia Wardatus Sholeha

**Affiliations:** 1 Department of Islamic Guidance and Counseling, Institut Agama Islam Negeri Langsa, Aceh, Indonesia; 2 Department of Guidance and Counseling, Universitas Negeri Malang, Malang, Indonesia; 3 Department of Nursing, Universitas Negeri Malang, Malang, Indonesia; 4 Department of Islamic Guidance and Counseling, Universitas Islam Negeri Raden Fatah, Palembang, Indonesia; 5 Department of Chemistry Education, Universitas Negeri Malang, Malang, Indonesia; 6 Department of Geography Education, Universitas Negeri Malang, Malang, Indonesia

Dear Editor,

We read with great interest the recent study by Cadmus et al. (2025) on the knowledge and perception of older adults toward end-of-life care and advance directives (AD) in Nigeria (Cadmus et al. 2025). The study provides crucial insights into a pressing yet often overlooked issue in low- and middle-income countries (LMICs): the lack of awareness and engagement in advance care planning (ACP) (Nakanishi et al. 2024). The findings highlight a critical gap that, if unaddressed, could lead to suboptimal end-of-life care and increased emotional and financial burdens for families. Given the growing elderly population globally, understanding cultural, social, and systemic barriers to ACP is imperative.

One of the most striking findings of this study is the low level of awareness regarding ACP, living wills, and power of attorney among Nigerian older adults, with fewer than 30% having considered any form of ACP. This suggests an urgent need for targeted educational interventions that respect sociocultural contexts. In many African societies, including Nigeria, discussing death remains taboo, with strong religious and familial influences dictating end-of-life decisions (Glyn-Blanco et al. 2023). This cultural dynamic often results in reluctance to engage in proactive healthcare planning, thereby limiting patient autonomy.

While Cadmus et al. underscore the role of religious leaders and media as primary sources of information, we propose that interventions should integrate these trusted community figures into ACP education and advocacy. A faith-based ACP awareness program, for instance, could align end-of-life planning with religious values, reassuring individuals that ACP is not an act of defiance against faith but rather a means of ensuring dignity and respect in healthcare decisions (Pohan et al. 2024a; Saputra et al. 2024). Additionally, intergenerational dialogues involving family members can facilitate open discussions about end-of-life preferences (Pohan and Astuti 2024). In many African traditions, healthcare decisions at the end of life are often made collectively rather than individually. Leveraging this cultural norm, structured family counseling sessions on ACP guided by healthcare professionals could enhance acceptance and participation in advance care planning (Kishino et al. 2022).

Beyond education, there is an evident need for policy-driven approaches that institutionalize ACP as part of routine geriatric care. National health policies should integrate ACP discussions into primary healthcare settings, ensuring that all older adults, particularly those with chronic illnesses, receive guidance on AD and end-of-life care options. This could be achieved through the development of standardized ACP toolkits tailored to Nigeria’s diverse cultural landscape, along with training programs for healthcare providers on how to initiate sensitive discussions about end-of-life preferences. Furthermore, digitizing ACP documentation and integrating it into electronic health records could enhance accessibility and ensure that healthcare providers respect patients’ documented preferences. Mobile health applications can also serve as platforms for older adults and their families to learn about ACP and complete digital living wills with guidance from medical professionals (Canny et al. 2023).

Nigeria is not alone in facing these challenges. Other LMICs, such as Rohingnya Refugees in Indonesia, India, and Brazil, have demonstrated the efficacy of community-based ACP programs that incorporate religious and traditional leaders into structured discussions on end-of-life care (Goins et al. 2024; Pohan et al. 2024b; Sawyer et al. 2021). Comparative research examining successful ACP adoption in similar sociocultural contexts could provide valuable insights for tailoring effective interventions in Nigeria and beyond.

The study by Cadmus et al. is a critical step in illuminating the gap in ACP awareness among older adults in Nigeria. However, moving from knowledge to action requires multi-sectoral collaboration among policymakers, healthcare providers, religious institutions, and the media. By developing culturally sensitive ACP initiatives and integrating them into the healthcare system, we can empower older adults to make informed decisions, improve end-of-life care quality, and alleviate the burden on families and caregivers. We urge researchers and policymakers to build on these findings through implementation research, testing interventions that blend medical, cultural, and technological solutions to bridge the ACP gap. Addressing this issue proactively will not only benefit Nigeria’s ageing population but also serve as a model for similar LMICs facing comparable challenges.
